# A New Approach of Oil Spill Detection Using Time-Resolved LIF Combined with Parallel Factors Analysis for Laser Remote Sensing

**DOI:** 10.3390/s16091347

**Published:** 2016-08-23

**Authors:** Deqing Liu, Xiaoning Luan, Jinjia Guo, Tingwei Cui, Jubai An, Ronger Zheng

**Affiliations:** 1 Optics and Optoelectronics Laboratory, Ocean University of China, Qingdao 266100, China; liudq1230@163.com (D.L.); luanxiaoning@ouc.edu.cn (X.L.); opticsc@ouc.edu.cn (J.G.); 2 First Institute of Oceanography, State Oceanic Administration, Qingdao 266100, China; cuitingwei@fio.org.cn; 3 Information Science & Technology College, Dalian Maritime University, Dalian 116026, China; jubaian@dlmu.edu.cn

**Keywords:** oil spill identification, time-resolved fluorescence, PARAFAC, laser remote sensing

## Abstract

In hope of developing a method for oil spill detection in laser remote sensing, a series of refined and crude oil samples were investigated using time-resolved fluorescence in conjunction with parallel factors analysis (PARAFAC). The time resolved emission spectra of those investigated samples were taken by a laser remote sensing system on a laboratory basis with a detection distance of 5 m. Based on the intensity-normalized spectra, both refined and crude oil samples were well classified without overlapping, by the approach of PARAFAC with four parallel factors. Principle component analysis (PCA) has also been operated as a comparison. It turned out that PCA operated well in classification of broad oil type categories, but with severe overlapping among the crude oil samples from different oil wells. Apart from the high correct identification rate, PARAFAC has also real-time capabilities, which is an obvious advantage especially in field applications. The obtained results suggested that the approach of time-resolved fluorescence combined with PARAFAC would be potentially applicable in oil spill field detection and identification.

## 1. Introduction

With the development of marine petroleum exploitation and transportation, fast growing oil spills have caused serious pollution to the oceanic environment and thus become an imminent problem. In order to deal with it, a rapid and reliable detection of oil spill contaminants is necessary. Fluorescence spectroscopy is one of the most useful methods in oil spill analysis as it allows quick and sensitive acquisition of polycyclic aromatic hydrocarbon (PHA) information in petroleum oil [1,2]. To identify spilled oils and link them to the known oil spill sources is important because it can provide evidence for prosecution and guide oil spill countermeasures [3,4]. Oil identification plays an important role in the oil spill disaster management [5,6].

In the study of oil spill identification, the approaches of fluorescence spectroscopy combined with pattern recognition and statistical methods including principal component analysis (PCA), artificial neural network (ANN), support vector machine (SVM), and parallel factors analysis (PARAFAC), etc. have been frequently reported [2,7,8]. PCA is one of the most widely used multivariate analysis methods [3], which has been used in many classic laser fluorosensor systems, such as Environment Canada’s Scanning Laser Environmental Airborne Fluorosensor (SLEAF), University of Oldenburg’s airborne laser fluorosensor (LFS), and Kuwait Institute for Scientific Research’s laser fluorosensor, etc. to process and analyze fluorescence spectra data for the oil classification of the three broad oil type categories: light refined, crude, or heavy refined [9,10,11,12,13]. In addition, the application of multivariate statistical methods, such as PARAFAC, to fluorescence excitation-emission matrixes (EEMs) of petroleum oils had good oil identification performances, though these methods are currently more laboratory based [14,15,16,17].

Time-resolved laser-induced fluorescence has also been applied to oil identification [18,19,20,21,22,23]. In the 1970s, the fluorescence decay studies by Rayner and Szabo have shown that measuring time decay together with the fluorescence spectra could allow for the identification of oil in remote sensing experiments [24], and Hegazi et al. have established a new method for remote fingerprinting of crude oil using time-resolved fluorescence, which can be used to discriminate crude oils of different grades [25]. These studies preliminarily evaluated the feasibility of time-resolved fluorescence in field detection of oil spills. It is well-known that PARAFAC is a multi-way decomposition method that generalizes PCA to higher order arrays, and the utmost advantage of this method is that it can recover pure spectra from multi-way spectra data and estimate components successively [26,27]. However, the application of PARAFAC for time-resolved fluorescence is hardly found in published literature, none other than Selli et al. [28] and Saito et al. [29] who had employed PARAFAC in analyzing the time-resolved fluorescence spectra of the mixtures of PAHs and the metal speciation, respectively.

In this paper, the application of time-resolved fluorescence technique in laser remote detection of oil spills has been demonstrated, and a new oil spill detection approach by the combination of time-resolved fluorescence and PARAFAC has also been established. The new method is expected to further improve the feasibility and accuracy of oil spill detection.

## 2. Apparatus and Methods

### 2.1. Time-Resolved Fluorescence Apparatus

**Figure 1:**
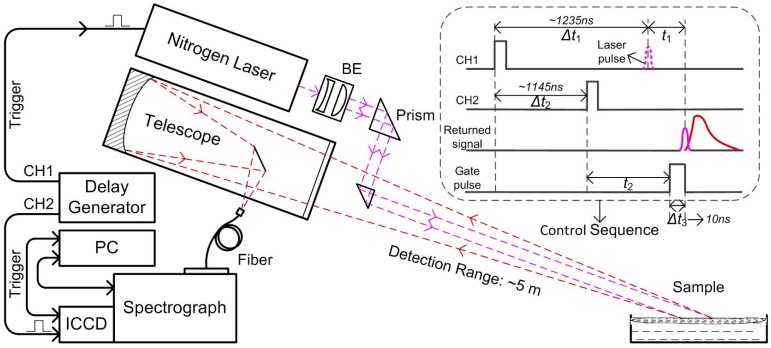
Schematic diagram of the time-resolved fluorescence experimental setup for laser remote sensing investigation of oil samples.

Figure 1 is the schematic diagram of the time-resolved fluorescence experimental setup specially developed for oil spill investigation. The experimental setup was established as a laser remote sensing system to meet the demand of shipborne field detection in the future, and had achieved about 5 m detection in the laboratory. As shown in Figure 1, the axis of the transmitter and receiver are coincident. A micro-pulse nitrogen laser (MNL100, LTB, Berlin, Germany) working at a wavelength of 337.1 nm is used as the excitation source. The laser pulse width is about 3 ns, and the laser pulse energy used in the investigation is 90 μJ, with a repetition of 10 Hz. To increase the interaction area between laser and oil and achieve remote detection, the laser beam is expanded and collimated before reaching the oil slick using a fused silica plano-concave lens (focal length: −50.8 mm) and plano-convex lens (focal length: 100 mm). The spot size of laser after beam expanding is about 10 mm. A transparent glass container was used as the sample cell. The seawater was put into the container, and the prepared oil samples were float on its surface. The oil film induced by the expanded laser beam emit fluorescence, and the returned optical radiation is collected by a Newtonian telescope (FirstScope, CELESTRON, Torrance, CA, USA) of which the aperture is 76 mm and focal ratio F/3.95, and then coupled into a 200 μm diameter optical fiber connected to a Czerny-Turner spectrometer (SR-303i, Andor, Oxford, UK). The spectrometer is equipped with a 150 L·mm^−1^ grating and an intensified charge coupled device (ICCD) camera (iStar DH720-18F-03, Andor, Oxford, UK), giving a broadband coverage from 338 to 736 nm with a spectral resolution of 0.1 nm. The determination of the spectral band relies on existing knowledge that the fluorescence response of crude oil when excited with an ultraviolet laser ranges from 400 to 650 nm, with peak centers in the 480 nm region [30].

In this system, as shown in Figure 1, the delay generator outputs two TTL signals (CH1 and CH2) with fixed delay time Δ*t*_2_ to accomplish the time sequence control of the laser and ICCD camera. CH1 and CH2 are the external trigger signal of the laser and ICCD, respectively. The embedded figure is the control sequence of the system, where Δ*t*_1_ is the fixed delay between external trigger signal and laser pulse, *t*_1_ is the propagation time from laser output to signal return, and *t*_2_, Δ*t*_3_ are the gate pulse delay and gate width of ICCD, respectively. In order to record complete time-resolved fluorescence spectra, the gate pulse of the image intensifier in ICCD was sequentially delayed according to the laser pulse by adjusting the gate pulse delay. In addition, to get higher signal to noise ratio (SNR) and time resolution, the time-resolved spectra were acquired with the parameters of 110 ns gate delay, 3 ns gate step and 10 ns gate width.

### 2.2. Sample Preparation

**Figure 2:**
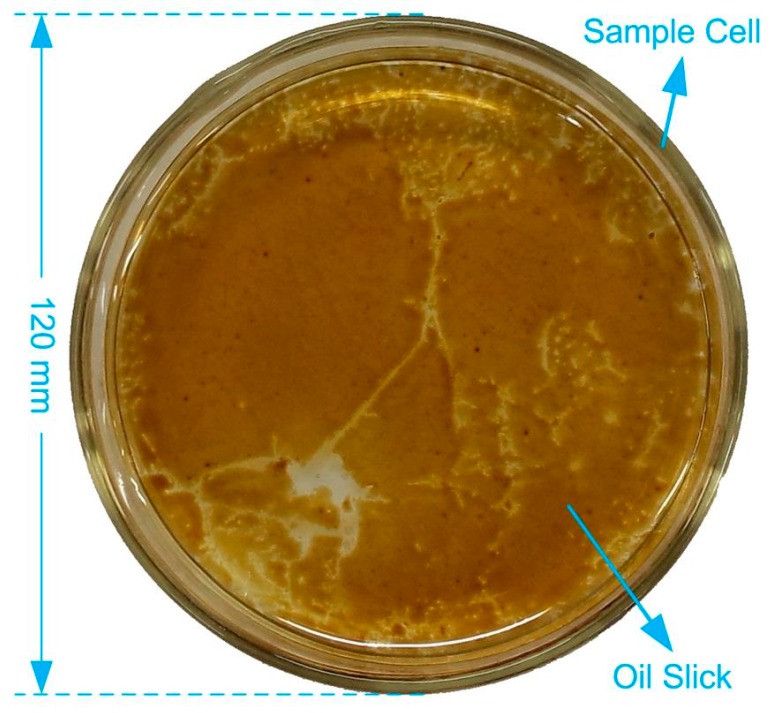
The picture of the prepared crude oil sample floating on the surface of sea water.

The oil samples used in the investigation included the refined oils (Gasoline and Diesel) and crude oil samples from four different wells (Bo601, Chengbei305, Shi138, and Zhengqi3) of Shengli oilfield. For convenience, the oil samples are coded as shown in Table 1, G as gasoline, D as diesel, and C1, C2, C3, and C4 as crude oils of Bo601, Chengbei305, Shi138, and Zhengqi3, respectively. The density and American Petroleum Institute (API) gravity of different oil samples are also listed in Table 1, and they reflect the grade of oil [31]. To prepare a floating oil slick for remote sensing investigation, some seawater was put into a transparent glass container with black tape stuck to the bottom to eliminate laser reflection. Then, the oil samples were released into the container to form a floating thin oil film on the surface of seawater, as shown in Figure 2. For the heavy weight crude oil samples of C3 and C4, additional melting procedure was needed because of their high viscosity. They were melted in hot water (~60 °C) before releasing into seawater.

### 2.3. Multi-Way Model: PARAFAC

As a multi-way decomposition method, PARAFAC decomposes N-way array into N loading matrices. In this work, the time-resolved fluorescence spectra of different oils were organized in a three-dimensional array **X**, where the number of oil samples was the first dimension, the number of emission wavelength the second, and the number of delay time the third. During the laser remote sensing investigation, the time-resolved fluorescence spectra were taken at five random detection positions on each of six oil floating samples to form 30 samplings in total. At each detection position, 15 emission spectra were obtained at different delay time $t_{2}$ from 110 ns to 152 ns with a 3 ns interval. From each emission spectrum, 553 emission intensities given by 553 pixels on the ICCD were selected with the wavelength coverage from 338 to 645 nm. In this way, the array **X** used in the PARAFAC model was set to be 30 × 553 × 15. The PARAFAC model of **X** can be written as:

(1) $$x_{ijk}=\sum_{f=1}^{F}a_{if}b_{jf}c_{kf}+e_{ijk}\;,\;i=1,\cdots ,30\;j=1,\cdots ,553\;k=1,\cdots ,15\;,$$

where $x_{ijk}$ is an element of **X**, which refers to the intensity of time-resolved fluorescence spectra for the *i*th sample, at emission wavelength *j* and delay time *k*. $a_{if}$, $b_{jf}$, $c_{kf}$ are elements of loading matrices A, B, C, respectively, and also the solutions of PARAFAC model. $e_{ijk}$ is the residual. The number of columns *F* in the loading matrices is the number of factors (components). In our analysis, the loading matrix A corresponds to the score of different oils with different factors, B and C correspond to the fluorescence spectra and temporal profiles, respectively.

**Figure 3:**
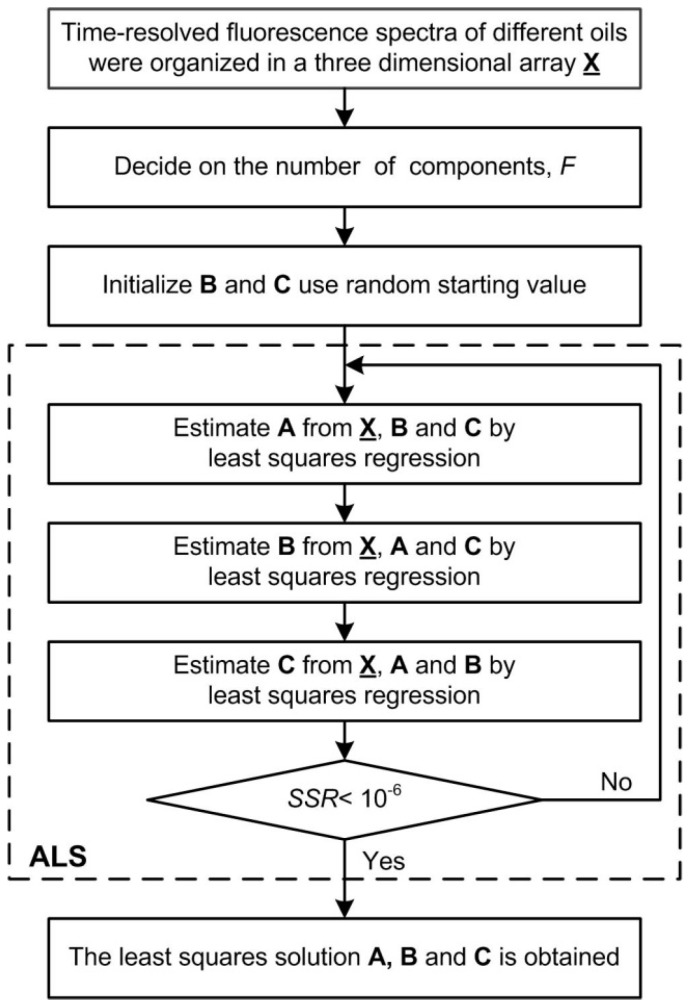
The flowchart of parallel factors analysis (PARAFAC) method used to decompose the time-resolved fluorescence spectra of oil samples.

The flowchart of PARAFAC method used to decompose the time-resolved fluorescence spectra of oil samples is shown in Figure 3. The solution to the PARAFAC model of Equation (1) was achieved by alternating least squares (ALS), and more detailed information can be found in [27]. Time-resolved fluorescence spectra of different oils were organized in a three-dimensional array **X**. The key point of PARAFAC ALS algorithm is the initialization of loading matrices B and C, which use random starting value, and estimation of the number of factors (components) *F*. The ALS algorithm will improve the fitness of the PARAFAC model. If the algorithm converges to the minimum of the sum of squared residual (SSR), the least squares solution A, B and C to the model would be obtained. A suitable stopping criterion is important, and a common criterion to use is to stop the iterations when the relative change in fit between two iterations is below a certain value (e.g., 10^−6^) [27]. The convergence criterion used in the work is shown as below:

(2) $$SSR=\sum_{i=1}^{I}\sum_{j=1}^{J}\sum_{k=1}^{K}e_{ijk}^{2}<1\times 10^{-6}.$$

## 3. Results and Discussion

### 3.1. Time-Resolved Fluorescence Spectra of Oils

**Figure 4:**
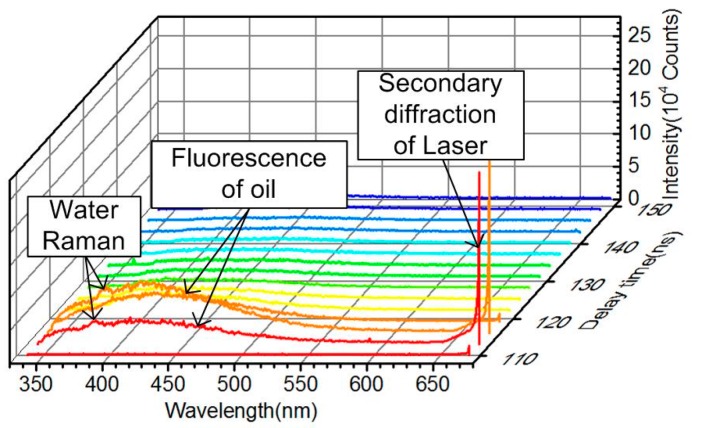
Typical returned signal of gasoline (G) taken by the laser remote sensing system.

**Figure 5:**
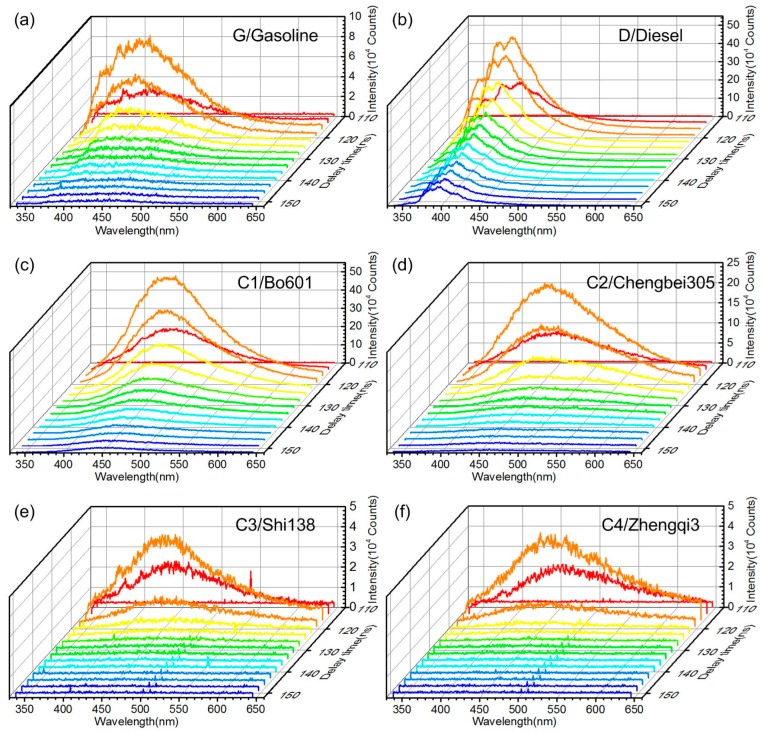
Typical time-resolved fluorescence spectra taken from gasoline (G), diesel (D) and 4 crude oil samples (C1–C4), (**a**,**b**) are the refined oil spectra of gasoline and diesel respectively, (**c**–**f**) are the crude oil spectra of B0601, Chengbei305, Shi138 and Zhengqi3 respectively.

Figure 4 is the typical returned signal of the laser remote sensing system taken from the gasoline samples. It can be seen that the returned signal mainly contains oil fluorescence and echo signals of laser secondary diffraction, and the water Raman signal can also be detected if the oil slick is thin enough. The typical time-resolved fluorescence spectra taken from six different oil samples are shown in Figure 5. It is obvious that the fluorescence peak of refined and crude oils are different, while the peaks are similar between different crude oils, and the fluorescence intensity of different crude oils varied with the grade of crude oil. The fluorescence peak location ($\lambda_{p}$) of each oil sample was listed in Table 1, and the peak fluorescent intensity of oil samples taken at five random detection positions are shown in Table 2. It reflects that the inhomogeneous distribution of oil slick on the water has influences on oil fluorescence signals.

### 3.2. Oil Identification Using PARAFAC

**Figure 6:**
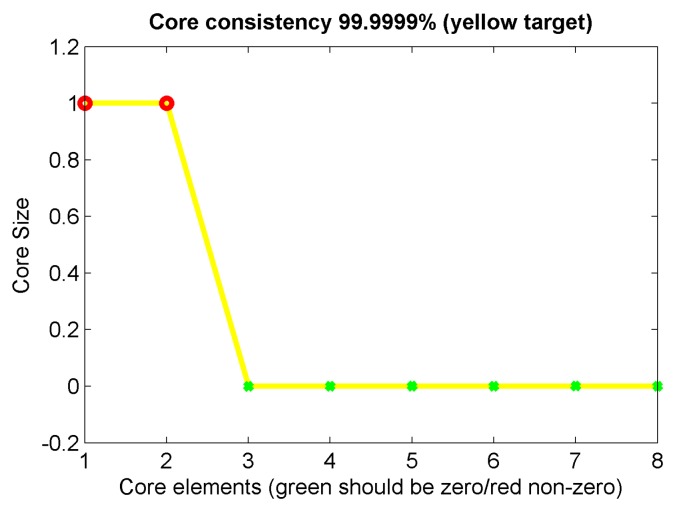
The result of core consistency obtained in the PARAFAC decomposition of original spectral data of different oils.

**Figure 7:**
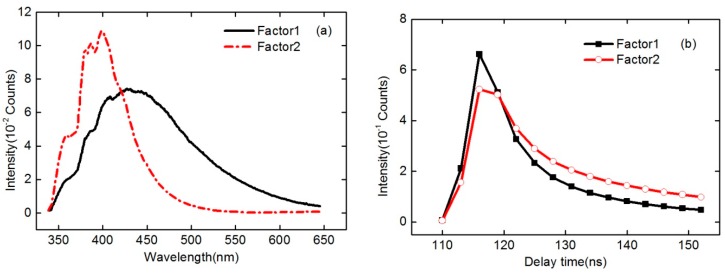
Intensities of the decomposed factors (*F* = 2) of PARAFAC as a function of emission wavelength (**a**) and delay time (**b**), respectively.

**Figure 8:**
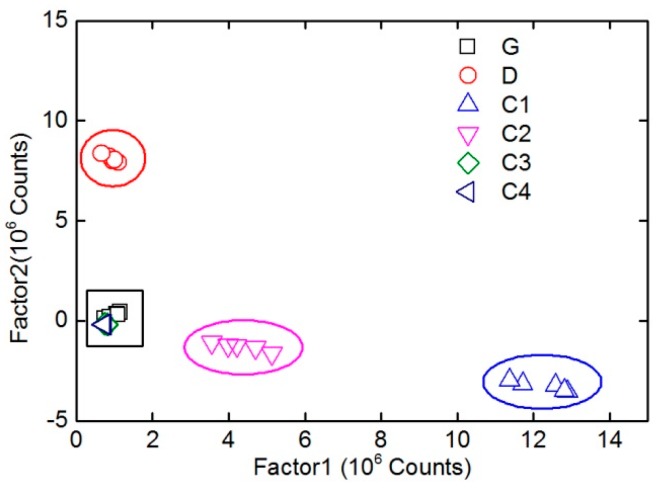
PARAFAC score scatter plot of six oil samples on two factors.

The original time-resolved fluorescence spectra of different oils without any preprocessing were decomposed and analyzed using the PARAFAC method. The first step of the method is to determine the number of factors (*F* in Equation (1)). Figure 6 is the result of core consistency obtained in the PARAFAC decomposition of original time-resolved spectral data of different oils. From Figure 6, it can be seen that for *F* > 2, the core consistency is close to 0. According to the core consistency and SSR in the PARAFAC model, the proper number of factors can be determined as two. The core consistency is a measure of “appropriateness” of a given PARAFAC model, and SSR is expected to stop decreasing when getting an appropriate number of factors because additional factors only explain random noises [29]. The results of the PARAFAC decomposition of time-resolved fluorescence spectra of all six oil samples with the number of factors *F* = 2 are shown in Figure 7 and Figure 8.

Figure 7a,b show the intensity of the decomposed factors (*F* = 2) of PARAFAC as a function of emission wavelength and delay time, respectively. As can be seen from Figure 7, two main components (factors) have been obtained from the PARAFAC decomposition of the time-resolved fluorescence spectra of the six oil samples. Compared with the spectral shape of original time-resolved fluorescence spectra of different oils, it is clear that the red dash dotted line in Figure 7a mainly corresponds to diesel, while the black solid line corresponds to the other oil samples in the experiment. Figure 8 is the PARAFAC score scatter plot of all six oil samples on the two factors. It has shown that D, C1, and C2 can be clearly distinguished, whereas G and two heavy crude oil samples (C3 and C4) can not. In addition, the spectral shapes and intensities of G, C3, and C4 are similar to each other from the original spectra of different oils.

As formerly mentioned, initial attempts to use PARAFAC method for original time-resolved fluorescence spectra of all oil samples without any preprocessing had produced unsatisfactory results, as some oil samples (such as G, C3, and C4) with similar fluorescence spectral shapes and intensities can not be well distinguished. The separation of oil samples in the factor space was seemingly related to the fluorescence intensity. However, the intensity of oil fluorescence was influenced by several factors, such as laser pulse energy, oil slick thickness, extinction coefficient of oil, and target distance, etc. In order to eliminate the effects of the changes in fluorescence intensity, more detailed analysis can be found in the following part.

**Figure 9:**
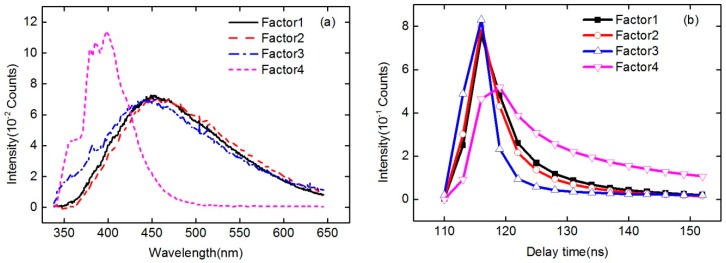
Intensities of the decomposed factors (*F* = 4) of PARAFAC as a function of emission wavelength (**a**) and delay time (**b**), respectively.

**Figure 10:**
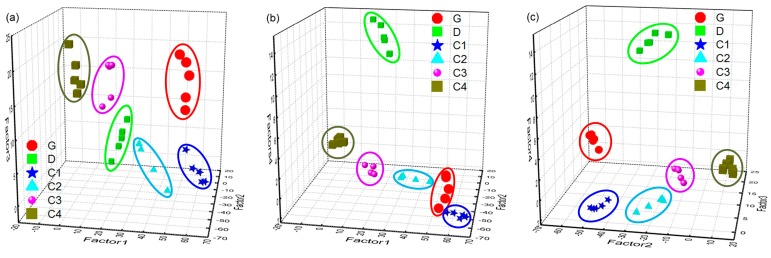
PARAFAC score scatter plots of six oil samples on four factors, (**a**) is the score scatter plot on factor1, factor2 and factor3, (**b**) is the score scatter plot on factor1, factor2 and factor4, (**c**) is the score scatter plot on factor2, factor3 and factor4.

The process and results of applying the PARAFAC method to normalized time-resolved fluorescence spectra of different oils are discussed in this part. Since the intensity of oil fluorescence was influenced by many factors, the time-resolved fluorescence spectra of all six oil samples were intensity-normalized, and then organized in the array **X** with its size still 30 × 553 × 15. Also according to the core consistency and SSR in the PARAFAC model, the number of factors *F* used in the PARAFAC model was determined to be 4, and the results of the PARAFAC decomposition of normalized time-resolved fluorescence spectra of all six oil samples with the number of factors *F* = 4 are shown in Figure 9 and Figure 10. Figure 9a,b are the intensity of the decomposed factors (*F* = 4) of PARAFAC as a function of emission wavelength and delay time, respectively. As can be seen from Figure 9, four main components (factors) were obtained from the PARAFAC decomposition of the normalized time-resolved fluorescence spectra of these oil samples. Compared with the spectral shape of original spectra of different oils, it can also be seen that the pink dashed line in Figure 9a mainly corresponds to diesel, the blue dash dotted line corresponds to the oil component which contains the water Raman signal, and the red dashed line as well as the black solid line mainly correspond to the heavy and light components, respectively. Figure 10 is the PARAFAC score scatter plots of all six oil samples on the four factors. As can be appreciated from the results, the oil samples of gasoline, diesel, and crude oils with different API gravities used in the experiment can be well classified. In addition, seemingly convincing oil identification results could be obtained by any three of the four components. As shown in Table 3, the correct separation rate in the factor space of oil samples used in the experiment proves that the coupling of intensity-normalized time-resolved fluorescence spectra with the PARAFAC method would be effective in oil identification.

### 3.3. Comparative Analysis of PARAFAC and PCA

Generally, laser fluorosensors were used to measure emission spectra of oil with the range gating in oil spill field detections, and the types of pollution oil can be real-time classified using the PCA algorithm [9,10,11,12]. In order to make a comparative analysis with the approach of PARAFAC coupled with time-resolved fluorescence, PCA was used to process the fluorescence emission spectra of the six oil samples investigated in the experiment.

**Figure 11:**
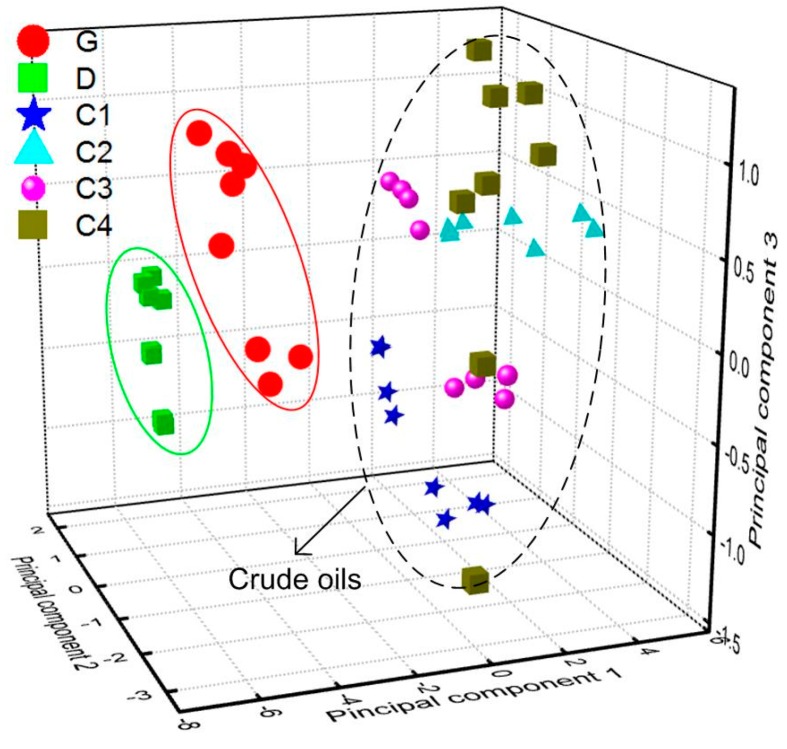
PCA score scatter plot of different oils on three principal components.

The fluorescence emission spectra were obtained at two of the five detection positions on each of the six oil samples, and four spectra were selected from the time-resolved fluorescence spectra in each detection position. As shown in Figure 5, the selected delay times were 113 ns, 116 ns, 119 ns, and 122 ns, respectively. In this way, the size of data array used in PCA was 48 × 553. When PCA is used to classify the oil types, the fluorescence spectra data has to be normalized for the oil’s fluorescence emission depending on various factors such as oil thickness and the distance from target, etc. [2]. Therefore, the fluorescence emission spectra data of all six oil samples used in PCA analysis also need intensity-normalized data preprocessing to eliminate the effects of the changes in fluorescence intensity. The processing results showed that only the first three principal components (PCs) contributed significantly to the data’s variance. The contribution of the three PCs were 83.2%, 7.9%, and 3% respectively, and the total contribution was more than 94%, hence oil separation was based on these three PCs. Figure 11 is the score scatter plot of different oils on the three PCs. It can be seen that the approach of PCA coupled with fluorescence emission spectra can differentiate refined and crude oils, but it is not available to the crude oil samples investigated in the experiment. As shown in Table 1, the fluorescence peak locations ($\lambda_{p}$) of these crude oils were quite close.

A comparative analysis is shown in Table 3, and the separation results of oil samples using PARAFAC and PCA algorithms for fluorescence spectra data of the oils are listed. It can be seen that both PCA and PARAFAC have real-time capabilities. PARAFAC needs determination of the appropriate number of factors before spectra data decomposition, and then the runtime of PARAFAC used to identify oil types is about four seconds. The computation could be faster by means of dedicated hardware resources. By comparison, PCA method could be commonly used to classify broad oil categories, whereas PARAFAC can not only differentiate refined and crude oil but also different crude oils from even the same oilfield with similar fluorescence spectral shapes and intensities. The coupling of intensity-normalized time-resolved fluorescence spectra with the PARAFAC method shows greater advantage in oil identification.

## 4. Conclusions

In this study, a new approach of oil spill detection for laser remote sensing was established by using time-resolved fluorescence combined with PARAFAC. A series of refined and crude oil samples were investigated using time-resolved fluorescence in conjunction with PARAFAC. The time resolved emission spectra of those investigated samples were taken by a laser remote sensing system at a laboratory basis with a detection distance of 5 m. Based on the intensity-normalized spectra, both refined and crude oil samples even from the same oilfield with similar fluorescence spectral shapes and intensities were well classified without overlapping, by the approach of PARAFAC with four parallel factors. PCA has also been operated as a comparison. It turned out that PCA operated well in classification of broad oil type categories, but with severe overlapping among the crude oil samples from different oil wells. Apart from the high correct identification rate, PARAFAC has also real-time capabilities, which is an obvious advantage especially in field applications. The obtained results suggested that the approach of time-resolved fluorescence combined with PARAFAC would be potentially applicable in oil spill field detection and identification. In future work, the investigation of oil spill field detection using time-resolved fluorescence coupled with PARAFAC method will be carried out.

## Figures and Tables

**Table 1:** Physical and spectral parameters of the oil samples used in the investigation.

Oil Samples		Density (kg·L^−1^)	API Gravity	Emission Peak Location $\lambda_{p}$ (nm)
Refined oil	Gasoline(G)	0.725	63.4	409.3
	Diesel(D)	0.835	38.0	401.0
Crude oil	Bo601(C1)	0.807	43.9	440.5
	Chengbei305(C2)	0.823	40.3	444.4
	Shi138(C3)	0.905	24.9	445.5
	Zhengqi3(C4)	0.979	13.1	449.4

**Table 2:** The peak fluorescent intensity of six oil samples taken at five random detection positions.

Detection Positions	Peak Fluorescent Intensity (10^4^ Counts)					
	Gasoline(G)	Diesel(D)	Bo601(C1)	Chengbei305(C2)	Shi138(C3)	Zhengqi3(C4)
Position1	8.4	50.8	53.1	23.1	3.9	3.9
Position2	8.0	48.7	53.8	18.8	4.1	3.8
Position3	4.6	46.3	56.1	17.6	4.3	3.9
Position4	5.7	49.6	48.3	17.3	4.5	4.0
Position5	7.6	48.7	55.6	15.7	4.5	3.7
Average	6.9	48.8	53.4	18.5	4.3	3.8
Relative standard deviation (RSD)	23.9%	3.4%	5.8%	15.2%	5.6%	3.3%

**Table 3:** Comparison of identification results using parallel factors analysis (PARAFAC) and principal component analysis (PCA) algorithms.

Oil Samples		PARAFAC			PCA		
		Spectra Number	Number of Overlapping	Runtime	Spectra Number	Number of Overlapping	Runtime
Refined oil	G	5	0	~4 s ^1^	8	0	~6 s
	D	5	0		8	0	
Crude oil	C1	5	1		8	6	
	C2	5	0		8	10	
	C3	5	0		8	15	
	C4	5	0		8	21	

^1^ Excluding the time used for testing the appropriate number of components in PARAFAC model.
